# Immunogenicity of half-dose versus full-dose *Leptospira* quadrivalent vaccine in dogs: a prospective randomized pilot study

**DOI:** 10.1093/jvimsj/aalag267

**Published:** 2026-11-13

**Authors:** Courtney Craig, Manoj K Pastey

**Affiliations:** College of Veterinary Medicine, Oregon State University, Corvallis, OR 97330, United States; Department of Biomedical Sciences, Oregon State University, Corvallis, OR 97330, United States

**Keywords:** antibody response, canine vaccination, dose response, leptospirosis, microscopic agglutination test

## Abstract

**Background:**

In clinical veterinary practice, incomplete vaccine administration can occur because of movement or partial injection loss; however, the immunologic consequences of reduced vaccine dosing are unknown.

**Hypothesis/Objectives:**

To compare antibody responses after administration of a half-dose versus a full-dose of a quadrivalent leptospirosis vaccine in dogs.

**Animals:**

Ten client-owned, healthy dogs (body weight range, 9-31 kg) with no history of leptospirosis vaccination.

**Methods:**

Prospective randomized pilot study. Dogs were assigned to receive either a full-dose (*n* = 5) or a half-dose (*n* = 5) of a quadrivalent inactivated leptospirosis vaccine. Serum samples were collected before vaccination and 4 weeks after vaccination. The primary outcome was post-vaccination microscopic agglutination test (MAT) titers against vaccine-associated serovars. Secondary outcomes included cross-reactive titers and associations with body weight.

**Results:**

All dogs were seronegative before vaccination. Dogs receiving the full-dose developed relatively uniform MAT responses against vaccine serovars, whereas responses in the half-dose group varied among individual dogs and serovars. Half-dose titers ranged from 1:100 to 1:3200, compared with 1:800 to 1:3200 in the full-dose group. Cross-reactive titers to *Leptospira bratislava* were detected in both groups, whereas all dogs remained seronegative for *Leptospira hardjo*. Within the half-dose group, dogs weighing < 50 lb had higher observed titers than dogs weighing ≥50 lb for several vaccine serovars.

**Conclusions and clinical importance:**

Reduced vaccine dosing resulted in variable antibody responses, with lower observed titers for some vaccine serovars, compared with full-dose vaccination, supporting adherence to recommended dosing to achieve consistent immunogenic responses in clinical practice.

## Graphical Abstract

**Graphical Abstract:**
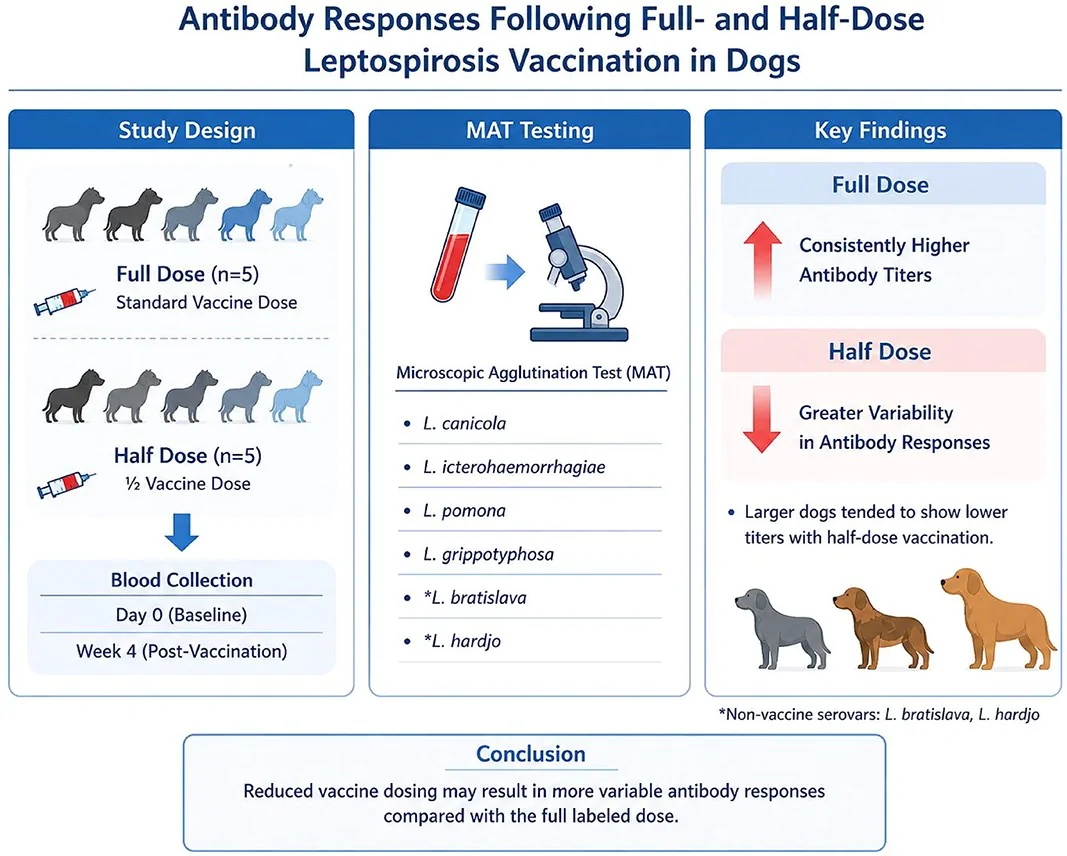

## Introduction

Leptospirosis is a globally distributed zoonotic disease caused by pathogenic *Leptospira* species and represents a major public health concern in both humans and animals.^1^ Dogs serve as both susceptible hosts and potential reservoirs, contributing to environmental contamination and zoonotic transmission.^2^ Clinical disease in dogs ranges from subclinical infection to severe multisystemic illness characterized by acute kidney injury, hepatic dysfunction, hemorrhagic syndromes, and death, with reported case mortality rates approaching 20% in clinically affected animals.^1^^,^^3^

Vaccination constitutes the cornerstone of leptospirosis prevention in dogs and reduces both disease severity and urinary shedding of the organism.^2^^,^^4^ Currently licensed canine leptospirosis vaccines are inactivated bacterins targeting multiple clinically relevant serovars, most commonly *Leptospira canicola, Leptospira icterohaemorrhagiae, Leptospira pomona,* and *Leptospira grippotyphosa.*^5^ However, in contrast to core viral vaccines, immunity induced by leptospirosis bacterins is relatively short-lived and requires regular booster administration.^4^

In clinical veterinary practice, partial vaccine delivery can occasionally occur due to movement during injection, leakage at the injection site, or equipment malfunction. Although revaccination is often recommended in such situations, there is limited information regarding the immunologic consequences of reduced-dose administration of leptospiral bacterin vaccines in dogs. Understanding the antibody response following incomplete vaccine delivery can help veterinarians make more informed clinical decisions when such situations arise. Previous investigations of reduced-dose vaccination have largely focused on viral vaccines, where partial doses can still elicit measurable antibody responses.^6^ However, leptospirosis bacterins differ substantially from viral vaccines in antigen composition and immunological properties, and no comparable evaluation has been conducted for reduced-doses of Leptospira vaccines.^2^

The microscopic agglutination test (MAT) is the reference standard serologic assay for detection of antibodies to *Leptospira.*^7^ Although MAT titers primarily reflect IgM responses and do not directly equate to protective immunity, they provide a useful comparative measure of humoral responses following vaccination.^8^ Cellular immune responses, which contribute significantly to protection against leptospiral infection, were not evaluated in this study. Because no previous studies have evaluated the immunological consequences of reduced-dose administration of leptospiral bacterin vaccines in dogs, an exploratory pilot study was conducted to generate preliminary data to inform the design of larger controlled investigations.

The objective of this study was to compare MAT antibody responses following administration of a half-dose versus a full-dose of a quadrivalent leptospirosis vaccine in adult dogs with no prior exposure to leptospirosis vaccination. A secondary objective was to assess the influence of body weight on immune responses among half-dose recipients.

## Materials and methods

### Study cohort

This prospective randomized study evaluated antibody responses following administration of reduced-dose leptospiral vaccination in dogs. Client-owned dogs presented for routine preventive care were considered eligible for enrollment. Ten adult dogs were enrolled in the study. Dogs ranged from 1 to 4 years of age and were deemed clinically healthy based on physical examination. None had a known history of leptospirosis vaccination or prior leptospiral infection. Baseline seronegativity for *Leptospira* antibodies was confirmed in all dogs using MAT. Although MAT seronegativity (<1:100) was used to confirm absence of detectable antibodies prior to vaccination, it is recognized that MAT detects circulating antibodies and does not completely exclude the possibility of asymptomatic renal carriage of *Leptospira* in clinically healthy dogs.

All procedures involving animals were approved by the University Institutional Animal Care and Use Committee (IACUC). Written informed consent was obtained from the owners of all client-owned dogs prior to enrollment, and owners were informed about the study procedures and sample collection.

Dogs were randomly assigned (simple randomization) to one of 2 groups (*n* = 5 per group):

- Full-dose group: received one full labeled dose of a quadrivalent inactivated leptospirosis vaccine (Nobivac® Lepto4^5^).
- Half-dose group: received one half of the labeled vaccine dose.

The vaccine contains inactivated *Leptospira* serovars *L. canicola, L. icterohaemorrhagiae, L. pomona,* and *L. grippotyphosa*. Vaccines were administered subcutaneously according to manufacturer recommendations, except for the reduced volume administered to the half-dose group.

A non-vaccinated control group was not included in this pilot study because the animals were client-owned dogs and administration of placebo injections was not considered ethically necessary. Instead, baseline seronegativity for Leptospira antibodies was confirmed in all dogs prior to vaccination to provide a reference for post-vaccination antibody responses. Because the study involved client-owned dogs living in natural environments, environmental exposure to *Leptospira* during the study period cannot be completely excluded. However, baseline MAT testing confirmed that all dogs were seronegative prior to vaccination, suggesting that the post-vaccination antibody responses observed were most likely associated with vaccine administration.

Whole blood (~3 mL) was collected before vaccination and 4 weeks post-vaccination, and serum samples were tested in duplicate by MAT at the University Veterinary Diagnostic Laboratory. Additional details regarding sample collection, processing, and laboratory procedures are provided in Appendix.

## Results

Ten client-owned dogs were enrolled in the study (Table 1). Median age was 3 years (range, 1-4 years), and median body weight was 23 kg (range, 9-31 kg). Breeds included Labrador Retriever (*n* = 2), mixed breed (*n* = 2), and other breeds (*n* = 6). Five dogs received the full vaccine dose, and 5 dogs received the half vaccine dose. All dogs were seronegative for all tested serovars prior to vaccination, confirming naïve status. Four weeks after vaccination, antibody responses were detected against vaccine-associated serovars. Post-vaccination MAT titers were measured against 6 Leptospira serovars, including 4 vaccine serovars and 2 non-vaccine serovars. Post-vaccination MAT titers were higher in the full-dose group for 3 of the 4 vaccine serovars based on group medians, whereas a wider range of titers was observed in the half-dose group (Table 2) and were also explored in relation to body weight (Figure 2).

**Table 1:** Characteristics of dogs enrolled in the study.

**Characteristic**	**Half-dose (*n* = 5)**	**Full-dose (*n* = 5)**
**Age (years), median (range)**	3 (2-4)	3 (1-4)
**Body weight (kg), median (range)**	20 (10-31)	27 (9-30)
**Female, *n* (%)**	2 (40%)	2 (40%)
**Male, *n* (%)**	3 (60%)	3 (60%)
**Neutered, *n* (%)**	5 (100%)	5 (100%)

**Table 2:** Post-vaccination MAT antibody titers in dogs receiving full-dose or half-dose leptospirosis vaccination.

**Serovar**	**Half-dose (*n* = 5), median (range)**	**Full-dose (*n* = 5), median (range)**
***L. canicola***	800 (100-800)	800 (800-3200)
***L. icterohaemorrhagiae***	800 (400-1600)	1600 (1600-3200)
***L. grippotyphosa***	1600 (1600-3200)	3200 (3200-3200)
***L. pomona***	800 (400-3200)	1600 (1600-3200)
***L. bratislava†***	800 (100-1600)	800 (800-3200)
***L. hardjo†***	0 (0-0)	0 (0-0)

MAT titers were measured 4 weeks after administration of a quadrivalent leptospirosis vaccine. Values are presented as median (range). Serovars *L. canicola*, *L. icterohaemorrhagiae*, *L. grippotyphosa*, and *L. pomona* are included in the vaccine formulation. *L. bratislava* and *L. hardjo* are non-vaccine serovars included for assessment of cross-reactive antibody responses.

### Group comparison (full-dose versus half-dose vaccination)

In the full-dose group, median titers against the 4 vaccine serovars ranged from 1:800 to 1:3200 (Table 2; Figure 1). In contrast, dogs receiving the half dose demonstrated greater variability in antibody responses. Three of 5 half-dose recipients (60%; 95% exact binomial CI, 14.7%-94.7%) had titers below 1:800 against at least one vaccine serovar.

**Figure 1:**
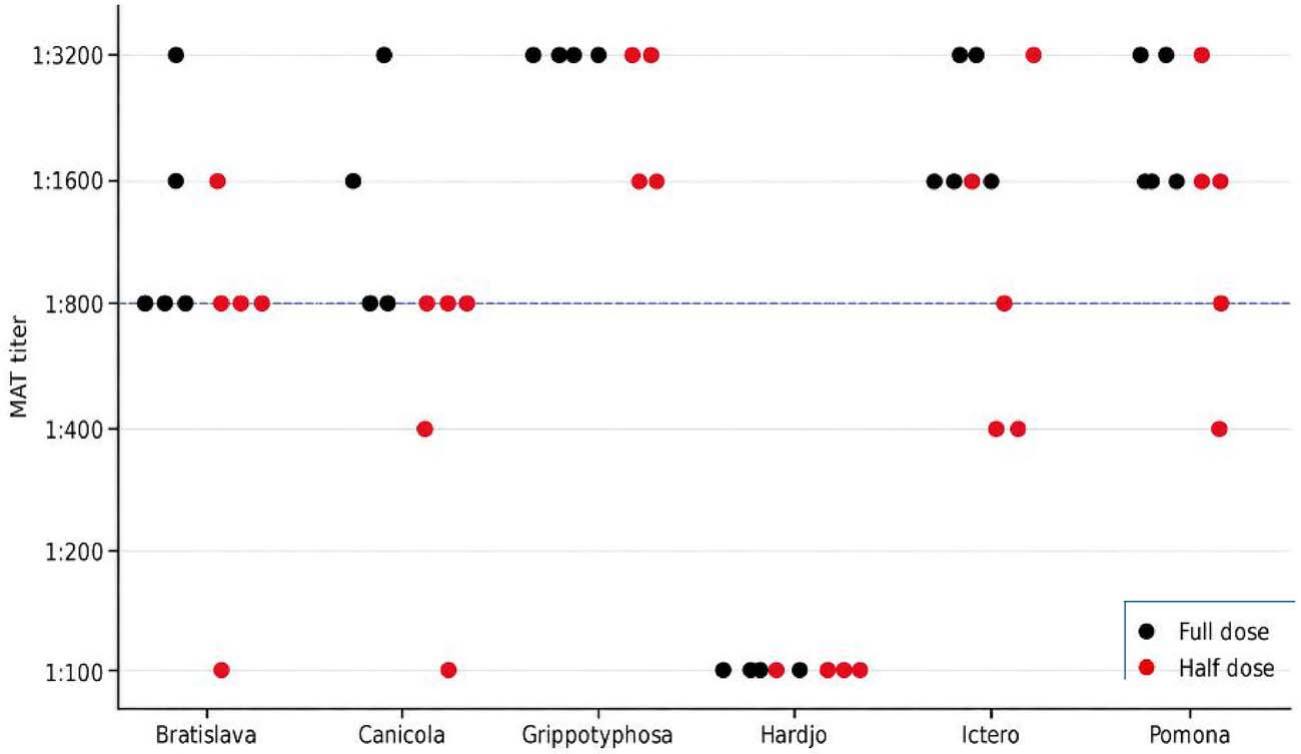
Serum antibody responses following full-dose and half-dose leptospirosis vaccination in dogs. Microscopic agglutination test (MAT) titers against *Leptospira* serovars *L. Bratislava, Leptospira canicola, L. grippotyphosa, L. hardjo, Leptospira icterohaemorrhagiae,* and *L. pomona* in dogs receiving either a full vaccine dose (*n* = 5) or half vaccine dose (*n* = 5). Each point represents an individual dog. The dashed horizontal line indicates a MAT titer of 1:800 and is included for visualization of higher antibody titers only; it does not represent a correlate of protective immunity. MAT titers are displayed on a logarithmic (log₂) scale to facilitate visualization of serial dilution titers. The figure is presented descriptively to illustrate response variability among individual dogs.

For *L. canicola*, titers in the half-dose group ranged from 1:100 to 1:800, whereas titers in the full-dose group ranged from 1:800 to 1:3200. Similar patterns were observed for *L. icterohaemorrhagiae* and *L. pomona*. All full-dose recipients developed high titers against *L. grippotyphosa* (1:3200), whereas half-dose recipients demonstrated a wider range of responses (1:1600-1:3200). These differences are presented descriptively and were not evaluated using formal hypothesis testing.

Cross-reactive antibody responses to *L. bratislava* were observed in both groups. All dogs remained seronegative for *L. hardjo*, which is not included in the vaccine formulation.

### Body weight and antibody responses

Within the half-dose group, observed MAT titers differed according to body-weight category (Figure 2). Dogs weighing < 50 lb had higher observed titers than dogs weighing ≥50 lb for *L. canicola, L. icterohaemorrhagiae,* and *L. pomona*. Titers against *L. grippotyphosa* were relatively high in both weight categories but remained variable among individual dogs. Because of the small number of dogs in each weight category, these observations are descriptive and should be evaluated in larger studies.

**Figure 2:**
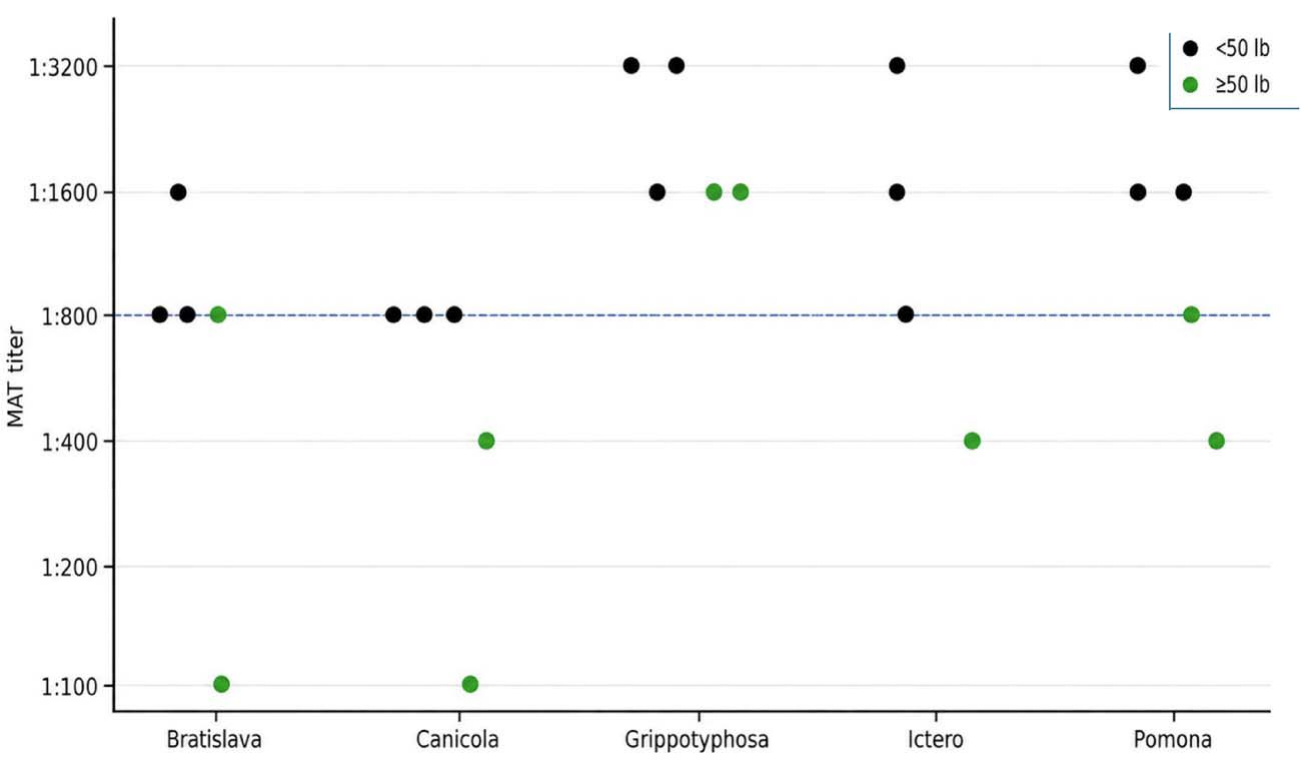
Serum antibody responses following half-dose leptospirosis vaccination in dogs stratified by body weight (<50 lb vs ≥ 50 lb). MAT titers in half-dose vaccine recipients stratified by body weight (<50 lb vs ≥50 lb). Each point represents an individual dog. The serovar *L. hardjo* is excluded because it is not included in the vaccine formulation and all dogs remained seronegative for this serovar. The dashed horizontal line indicates a MAT titer of 1:800 and is included for visualization of higher antibody titers only; it does not represent a correlate of protective immunity. MAT titers are displayed on a logarithmic (log₂) scale to facilitate visualization of serial dilution titers. The figure is presented descriptively to illustrate response variability among individual dogs.

## Discussion

This study demonstrated that administration of a half dose of a quadrivalent leptospirosis vaccine resulted in greater variability in antibody responses compared with administration of the full labeled dose. Although half-dose vaccination elicited measurable antibody responses, the magnitude of the response varied among individual dogs and serovars. From a clinical perspective, incomplete vaccine administration occasionally occurs in veterinary practice because of patient movement during injection or partial loss of vaccine volume. The findings of this study suggest that partial vaccine dosing could result in variable antibody responses and potentially reduced immunogenicity. When incomplete vaccine delivery is suspected, revaccination with the full recommended dose could be considered to support adequate immune stimulation.

These findings contrast with reports of reduced-dose viral vaccination and likely reflect fundamental immunological differences between viral antigens and inactivated bacterial bacterins.^2^^,^^6^ Leptospiral vaccines rely on whole-cell antigens and require sufficient antigen mass to induce effective humoral immunity; therefore, reduction in antigen quantity due to incomplete vaccine administration could reduce vaccine immunogenicity.^4^

Interpretation of MAT titers warrants caution, as the assay primarily reflects IgM antibodies and does not directly assess IgG-mediated memory or cellular immunity.^7^^,^^8^ In addition, MAT is a semi-quantitative assay that relies on visual assessment of agglutination, and differences of a single dilution (e.g. 1:800 versus 1:1600) can fall within normal assay variability. Consequently, MAT titers should not be interpreted as direct correlates of protective immunity, and the findings of this study should be interpreted strictly as differences in measurable antibody responses rather than evidence of vaccine efficacy. Nevertheless, MAT provides a useful comparative metric for assessing relative immunogenicity between dosing regimens.

In the present study, a MAT titer of 1:100 represents the lowest dilution at which antibodies can be detected using the diagnostic laboratory protocol employed. Titers below this level were considered seronegative. The classification of titers in this study was used only for descriptive comparison of antibody responses and does not represent established protective thresholds. MAT titers following leptospiral vaccination are often low and short-lived. Experimental challenge studies have demonstrated that vaccinated dogs can remain protected against infection even in the absence of detectable MAT titers, indicating that MAT is not a reliable correlate of protective immunity.^2^^,^^4^^,^^8^

An unexpected observation was the detection of cross-reactive antibody responses to *L. bratislava*, suggesting partial antigenic overlap among leptospiral serovars. These findings should be interpreted as serologic cross-reactivity rather than cross-protective immunity, as MAT cross-reactions are common during early antibody responses. In contrast, the absence of responses to *L. hardjo* reinforces the serovar-specific nature of leptospiral immunity and supports the continued use of multivalent vaccine formulations.^2^

An additional observation was that larger dogs receiving a half vaccine dose appeared to develop lower or more variable antibody responses than smaller dogs. This finding is biologically plausible and suggests that a reduced fixed dose could result in underdosing larger animals. However, the study was not designed to determine weight-adjusted vaccine dosing strategies.

Several limitations should be acknowledged. First, the study included a small number of animals and was designed as an exploratory pilot study intended to generate hypotheses rather than provide definitive conclusions. Second, a non-vaccinated control group was not included because the study involved client-owned dogs and placebo administration was not considered ethically necessary. However, all dogs were confirmed seronegative before vaccination, providing a baseline reference for post-vaccination antibody responses. Because the study involved client-owned dogs living in natural environments, environmental exposure to *Leptospira* during the study period cannot be completely excluded. However, baseline MAT testing confirmed that all dogs were seronegative prior to vaccination, suggesting that the antibody responses observed were most likely associated with vaccine administration. Third, cellular immune responses and protective efficacy were not evaluated. Additionally, although dogs were seronegative prior to vaccination, asymptomatic carrier status cannot be completely excluded because MAT detects circulating antibodies rather than persistent renal colonization. Fourth, only a single commercially available quadrivalent leptospirosis vaccine was evaluated; therefore, the findings cannot necessarily be generalized to other leptospiral vaccine formulations. Finally, all dogs received only a single vaccine administration and were evaluated 4 weeks later. Because leptospirosis vaccination protocols typically include booster vaccination in previously unvaccinated dogs, it remains unknown whether subsequent full-dose or half-dose booster vaccinations would result in similar antibody responses.

In conclusion, this study directly evaluates reduced-dose immunogenicity of a quadrivalent leptospirosis vaccine in dogs. These findings are consistent with current recommendations for administration of the full labeled dose of leptospirosis vaccines and caution against reduced-dose administration.

## Data Availability

The raw data supporting the conclusions of this article will be made available by the corresponding author.

## Off-label antimicrobial declaration

Authors declare no off-label use of antimicrobials.

## Institutional animal care and use committee or other approval declaration

The animal study was reviewed and approved by the University Institutional Animal Care and Use Committee (IACUC), Protocol No. 2023-0393. Written informed consent was obtained from the owners of all client-owned dogs prior to enrollment, and owners were informed about the study procedures and sample collection.

## Appendix: Detailed Materials and Methods

### Sample collection and laboratory testing

Whole blood (~3 mL) was collected before vaccination and 4 weeks post-vaccination. Samples were allowed to clot and centrifuged to obtain serum and stored at −20 °C until testing by the University Veterinary Diagnostic Laboratory.

Antibody titers were measured using the MAT against 6 leptospiral serovars, including those contained in the vaccine as well as selected additional serovars used for diagnostic screening. The MAT was performed using serial dilution of serum samples in duplicate with standard laboratory procedures.

Primary outcome measures included post-vaccination antibody titers and changes in antibody titers relative to baseline values. Because of the pilot nature of the study and the small sample size (*n* = 5 per group), data were analyzed descriptively.

### Microscopic agglutination test

Each serum sample was tested in duplicate using MAT according to standard diagnostic laboratory protocols. Live cultures of *Leptospira* serovars were used as antigens. Serum samples were serially diluted and incubated with each serovar-specific antigen preparation. Agglutination was assessed by dark-field microscopy.

Agglutination of ≥50% of organisms was considered a positive reaction at a given dilution, and the highest serum dilution meeting this criterion was recorded as the endpoint titer. Endpoint titers were determined by visual assessment of agglutination by experienced laboratory personnel, and concordant results were recorded as the final titer.

Samples were tested against six serovars: *L. bratislava, L. canicola, L. grippotyphosa, L. hardjo, L. icterohaemorrhagiae,* and *L. pomona*.

### Interpretation of MAT titers

Titers were interpreted as follows:

<1:100, seronegative;

1:100-1:800, suggestive of a detectable antibody response;

≥1:800, suggestive of a higher magnitude antibody response.

In the present study, a MAT titer of 1:100 represents the minimum dilution at which antibodies can be detected using the diagnostic laboratory protocol employed. Titers ≥1:100 were interpreted as evidence of detectable antibodies, whereas titers below this level were considered seronegative. MAT titers were used only as a comparative measure of humoral antibody responses between groups and should not be interpreted as correlates of protective immunity.

### Data analysis and visualization

Because of the pilot nature of the study and the small sample size (*n* = 5 per group), data were analyzed descriptively. For descriptive categorical outcomes, results are reported as the number and proportion of dogs meeting the specified criterion, with 2-sided 95% exact binomial (Clopper–Pearson) confidence intervals. Individual MAT titers were summarized by serovar and vaccine group. Median and range were used to describe antibody responses. Results are presented to illustrate patterns of response and variability among individual dogs rather than to test specific hypotheses.

Titers were visualized using log₂-scaled scatter plots with individual animals represented as discrete data points. Additional visualization was performed within the half-dose group to explore the relationship between body weight and antibody responses. No formal hypothesis testing was performed.

## Abbreviation

MAT: microscopic agglutination test
